# Physical multimorbidity, suicidal ideation, and suicide attempts among adults aged ≥50 years from low‐ and middle‐income countries

**DOI:** 10.1002/gps.5873

**Published:** 2023-01-22

**Authors:** Lee Smith, Jae Il Shin, Guillermo F. López Sánchez, Karel Kostev, Louis Jacob, Mark A. Tully, Laurie Butler, Yvonne Barnett, Nicola Veronese, Pinar Soysal, Adel S. Abduljabbar, Josep Maria Haro, Ai Koyanagi

**Affiliations:** ^1^ Centre for Health, Performance, and Wellbeing Anglia Ruskin University Cambridge UK; ^2^ Department of Pediatrics Yonsei University College of Medicine Seodaemun‐gu Seoul Korea; ^3^ Division of Preventive Medicine and Public Health Department of Public Health Sciences School of Medicine University of Murcia Murcia Spain; ^4^ Philipps University of Marburg Marburg Germany; ^5^ Research and Development Unit Parc Sanitari Sant Joan de Déu, CIBERSAM, ISCIII, Sant Boi de Llobregat Barcelona Spain; ^6^ Faculty of Medicine University of Versailles Saint‐Quentin‐en‐Yvelines Montigny‐le‐Bretonneux France; ^7^ School of Medicine Ulster University Londonderry UK; ^8^ Geriatrics Section Department of Internal Medicine University of Palermo Palermo Italy; ^9^ Department of Geriatric Medicine Faculty of Medicine Bezmialem Vakif University Istanbul Turkey; ^10^ King Saud University Riyadh Saudi Arabia; ^11^ ICREA Barcelona Spain

**Keywords:** adults, epidemiology, low‐ and middle‐income countries, multimorbidity, suicidal ideation, suicide attempts

## Abstract

**Objectives:**

The association between physical multimorbidity and suicidal ideation or suicide attempts among older adults from low‐ and middle‐income countries (LMICs) is largely unknown. We aimed to assess this association as well as its mediators using nationally representative data from six LMICs.

**Methods:**

Cross‐sectional, community‐based data from the Study on Global Aging and Adult Health were analyzed. A total of 11 chronic physical conditions were assessed. Self‐reported information on past 12‐month suicidal ideation and suicide attempts was also collected. Multivariable logistic regression and mediation analyses were conducted.

**Results:**

The final sample consisted of 34,129 adults aged ≥50 years (mean [SD] age 62.4 (16.0) years; maximum age 114 years; 52.1% females). In the overall sample, physical multimorbidity was associated with increased odds for suicidal ideation (OR = 2.99; 95% CI = 2.06–4.34) and suicide attempts (OR = 2.79; 95% CI = 1.58–4.95), with the association being stronger in males than females. The association between multimorbidity and suicidal ideation or suicide attempts was largely mediated by pain/discomfort (mediated% 33.3%–44.2%), sleep/energy (28.2%–33.8%), and mobility limitations (26.6%–34.8%).

**Conclusions:**

Physical multimorbidity among older adults in LMICs was associated with a substantially increased risk for suicidal ideation and suicide attempts. Addressing the identified mediators in people with physical multimorbidity may aid in the prevention of suicidal ideation and suicide attempts.

## Key points

- 34,129 adults aged ≥50 years (mean [SD] age 62.4 (16.0) years; 52.1% females).
- Physical multimorbidity associated with increased risk for suicidal ideation.
- Physical multimorbidity associated with increased risk for suicide attempts.

## 1 INTRODUCTION

Suicide is death caused by injuring oneself with the intent to die, while a suicide attempt is self‐harm with an intent to die, but which does not result in death.^1^ Suicidal ideation is a term used to describe a range of contemplations, wishes, and preoccupations with death and suicide,^2^ and usually precedes a suicide attempt. Approximately 700,000 people die from suicide each year, of which 77% occur in low‐ and middle‐income countries (LMICs).^3^ Although suicide attempts are more frequent among adolescents and young adults, older people show the highest suicide rate in almost all countries.^4^ Moreover, the rate of suicide increases with age among people older than 60 years.^5^ Importantly, for every suicide, there are many more people who attempt suicide, and a prior suicide attempt is the single most important risk factor for suicide in the general population.^3^ Considering these issues, it is of utmost importance to identify risk factors of suicidal ideation and suicide attempts to aid in the development of targeted interventions among older adults in LMICs. While there is a myriad of identified determinants of suicidal behavior and thoughts, one potentially important but understudied risk factor is that of physical multimorbidity.

Physical multimorbidity may be defined as the presence of two or more long‐term physical health conditions.^6^ Physical multimorbidity may increase risk for suicidal ideation and suicide attempts via, for example, social exclusion, functional limitation, perceived burdensomeness, and economic burden.^7^ In a recent meta‐analysis consisting of 13 studies exclusively from high‐income countries on physical multimorbidity and suicidal ideation, it was found that physical multimorbidity was associated with a 1.8 times greater odds for suicidal ideation.^7^ More recently, a study conducted in China found among 3242 adults aged 60 years and older that those with two or more chronic physical conditions experienced significantly higher risk of suicidal ideation and suicidal plans. Moreover, the association between multimorbidity and suicidal ideations/plans was partially mediated by psychological distress, of which, the mediating effect of psychological distress accounted for 31.7% and 25.5% of the total effect, respectively.^8^ Although there are some studies on physical multimorbidity and suicidal ideation, to the best of our knowledge, there is only one published study on physical multimorbidity and suicide attempts to date. Specifically, this large study from the United States (*n* = 15,311) found that an increasing number of physical health conditions was associated with higher odds for suicide attempts; compared to those with no physical conditions, individuals with ≥4 physical illnesses had 4.39 times higher odds for suicide attempts.^9^

However, the main limitation of the existing literature is that all studies were conducted in high‐income countries, with the exception of one study from China which focused on suicidal ideation and plans.^8^ This is a major research gap as results from high‐income countries are unlikely to be generalizable to LMICs owing to difference in availability of health care for both mental health and physical health, and different disease profiles. Moreover, to date, mediators in the multimorbidity/suicidal ideation and suicide attempt relationships has been little studied, with the exception of the one previous discussed study from China that focused on psychological distress.^8^ It is important to investigate other potential mediating variables as there is the possibility that these can be targeted to mitigate suicidal ideation and suicide attempts among those with multimorbidity. Finally, studies focusing exclusively on older adults are scarce, despite the fact that suicides and multimorbidity are known to be most common in this population.

Given this background, the aim of the present study was to examine associations of multimorbidity with suicidal ideation and suicide attempts in a sample of 34,129 adults aged ≥50 years from six LMICs (China; Ghana; India; Mexico; Russia; South Africa). A further aim was to investigate the potential mediators of these associations. We hypothesized that those with multimorbidity will have higher odds of suicidal ideation and suicide attempt compared to those without this condition, and that this association will be partly mediated by social, psychological, and physical factors.

## 2 METHODS

Data from the Study on Global Aging and Adult Health (SAGE) were analyzed. This survey was conducted in China, Ghana, India, Mexico, Russia, and South Africa between 2007 and 2010. Based on the World Bank classification at the time of the survey, all these countries were LMICs. Details of the survey methodology have been published elsewhere.^10^ Briefly, in order to obtain nationally representative samples, a multistage clustered sampling design method was used. The sample consisted of adults aged ≥18 years with oversampling of those aged ≥50 years. Trained interviewers conducted face‐to‐face interviews using a standard questionnaire. Standard translation procedures were undertaken to ensure comparability between countries. The survey response rates were: China 93%; Ghana 81%; India 68%; Mexico 53%; Russia 83%; and South Africa 75%. Sampling weights were constructed to adjust for the population structure as reported by the United Nations Statistical Division. Ethical approval was obtained from the WHO Ethical Review Committee and local ethics research review boards. Written informed consent was obtained from all participants.

### 2.1 Suicidal ideation and suicide attempts

Information on suicidal ideation and suicide attempts were assessed in the same way as in previous SAGE publications,^11^, ^12^, ^13^ using an adapted version of the depression module of the WHO Composite International Diagnostic Interview.^14^ Those who screened positive in the depression module were further asked about suicidal thoughts and behavior. A positive screen referred to having at least one of the three following conditions for more than 2 weeks in the past 12 months: sadness, loss of interest, or low energy. Suicidal ideation was assessed by the question “Did you think of death, or wish you were dead?” and suicide attempts by the question “During this period, did you ever try to end your life?” with “yes” and “no” answer options.^11^, ^12^

### 2.2 Chronic physical conditions and physical multimorbidity

We included all 11 chronic physical conditions for which data were available in the SAGE. *Chronic back pain* was defined as having had back pain everyday during the last 30 days. Respondents who answered affirmatively to the question “Have you lost all of your natural teeth?” were considered to have *edentulism*. The participant was considered to have *hearing problems* if the interviewer observed this condition during the survey. *Hypertension* was defined as having at least one of the following: systolic blood pressure ≥140 mmHg; diastolic blood pressure ≥90 mmHg; or self‐reported diagnosis. *Visual impairment* was defined as having extreme difficulty in seeing and recognizing a person that the participant knows across the road.^15^ *Diabetes* and *stroke* were solely based on lifetime self‐reported diagnosis. For other conditions, the participant was considered to have the condition in the presence of either one of the following: self‐reported diagnosis; or symptom‐based diagnosis based on algorithms. We used these algorithms, which have been used in previous studies using the same dataset, to detect undiagnosed cases.^16^, ^17^ Specifically, the validated Rose questionnaire was used for *angina*,^18^ and other previously validated symptom‐based algorithms were used for *arthritis*, *asthma*, and *chronic lung disease*.^16^ Further details on the definition of chronic physical conditions can be found in Table A2 (Appendix). The total number of chronic physical conditions was calculated, and multimorbidity was defined as ≥2 chronic physical conditions, in line with previously used definitions.^17^ The number of chronic conditions was also categorized as 0, 1, 2, 3, and ≥4 in some analyses.

### 2.3 Mediators

We focused on unemployment, cognition, anxiety, perceived stress, sleep/energy, mobility, loneliness, social participation, disability, poor self‐related health, and pain/discomfort based on the possibility that they can be the consequence of multimorbidity, while they can also lead to suicidal behavior.^19^, ^20^, ^21^, ^22^, ^23^ Unemployment referred to not being engaged in paid work ≥2 days in the last 7 days. Sleep/energy, mobility, cognition, perceived stress, and pain/discomfort were assessed with two questions each. The actual questions can be found in supplementary Table A1. Each item was scored on a five‐point scale ranging from “none” to “extreme/cannot do” (sleep/energy, mobility, cognition, pain/discomfort) or “never” to “very often” (perceived stress). For each separate health status, we used factor analysis with polychoric correlations to obtain a factor score which was later converted to scores ranging from 0 to 100 with higher values representing worse health function.^24^ Those who claimed to have severe/extreme problems with worry or anxiety in the past 30 days were considered to have anxiety.^24^ Loneliness was assessed with the question “Did you feel lonely for much of the day yesterday?” with answer options “yes” or “no”. Disability was assessed with six questions on the level of difficulty in conducting standard basic activities of daily living (ADL) in the past 30 days (washing whole body, getting dressed, moving around inside home, eating, getting up from lying down, and using the toilet).^25^ Those who answered severe or extreme/cannot do to any of the six questions were considered to have disability.^24^ Following a previous SAGE publication,^26^ a social participation scale was created based on nine questions on the participant's involvement in community activities in the past 12 months (e.g., attended religious services, club, society, union etc) with answer options “never (coded = 1)”, “once or twice per year (coded = 2)”, “once or twice per month (coded = 3)”, “once or twice per week (coded = 4)”, and “daily (coded = 5)”. The answers to these questions were summed and converted to a scale ranging from 0 to 100 with higher scores indicating higher levels of social participation. Those who answered “bad” or “very bad” to the question “In general, how would you rate your health?” were considered to have poor self‐rated health.

### 2.4 Control variables

The selection of control variables was based on past literature,^27^ and included age, sex, years of education received, wealth quintiles based on income, alcohol use in the past 30 days, and smoking (never, past, current).

### 2.5 Statistical analysis

The statistical analysis was done with Stata 14.2 (Stata Corp LP, College station, Texas). The analysis was restricted to those aged ≥50 years. The difference in sample characteristics were tested by Chi‐squared tests and Student's *t*‐tests for categorical and continuous variables, respectively. Multivariable logistic regression analysis was done to assess the association between number of chronic conditions, or multimorbidity (i.e., ≥2 chronic conditions) (exposures) and suicidal ideation or suicide attempts (outcomes). These analyses were also stratified by sex. Furthermore, test of trend was conducted by including the number of chronic conditions in the model as a continuous variable rather than a categorical variable. In order to test whether the strength of the association between multimorbidity and suicidal ideation or suicide attempts differs by sex, we conducted interaction analysis by including the product terms of sex X multimorbidity in the models. We also conducted analysis with the individual chronic conditions as the exposure variable including all 11 chronic conditions simultaneously in the model. In order to assess the between country‐heterogeneity in the association between multimorbidity and suicidal ideation, we conducted country‐wise analysis and calculated the Higgin's *I* ^*2*^, which represents the degree of heterogeneity that is not explained by sampling error with values of 25%, 50%, and 75% often being considered as low, moderate, and high levels of heterogeneity.^28^ Overall estimates were obtained based on country‐wise estimates by meta‐analysis with fixed effects. Country‐wise analysis with suicide attempts as the outcome could not be conducted because stable estimates could not be obtained due to the small number of suicide attempts in each country.

Next, in order to gain an understanding of the extent to which various factors (i.e., unemployment, cognition, anxiety, perceived stress, sleep/energy, mobility, pain/discomfort, loneliness, social participation, disability, self‐rated health) may explain the relation between multimorbidity and suicidal ideation or suicide attempts, we conducted mediation analysis. We used the *khb* (Karlson Holm Breen) command in Stata^29^ for the mediation analysis. This method can be applied in logistic regression models and decomposes the total effect (i.e., unadjusted for the mediator) of a variable into direct and indirect effects. Using this method, the percentage of the main association explained by the mediator can also be calculated (mediated percentage). Each potential mediator was included in the model individually.

All regression analyses including the mediation analysis were adjusted for age, sex, education, wealth, alcohol consumption, smoking, and country with the exception of the sex‐ and country‐stratified analyses which were not adjusted for sex and country, respectively. Adjustment for country was done by including dummy variables for each country in the model as in previous SAGE publications.^30^, ^31^ The sample weighting and the complex study design were taken into account in all analyses. Results from the regression analyses are presented as odds ratios (ORs) with 95% confidence intervals (CIs). The level of statistical significance was set at *p* < 0.05.

## 3 RESULTS

**FIGURE 1:**
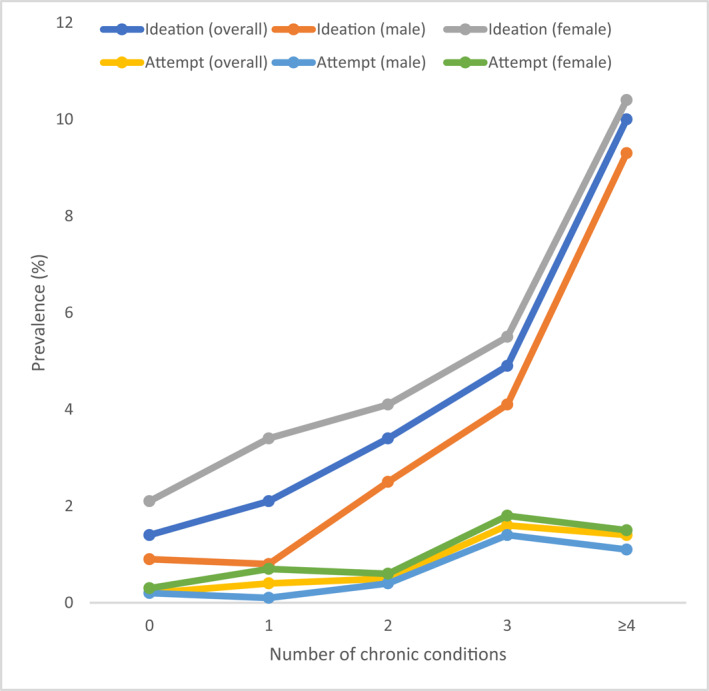
Prevalence of suicidal ideation and suicide attempts by number of chronic conditions (overall and by sex).

**FIGURE 2:**
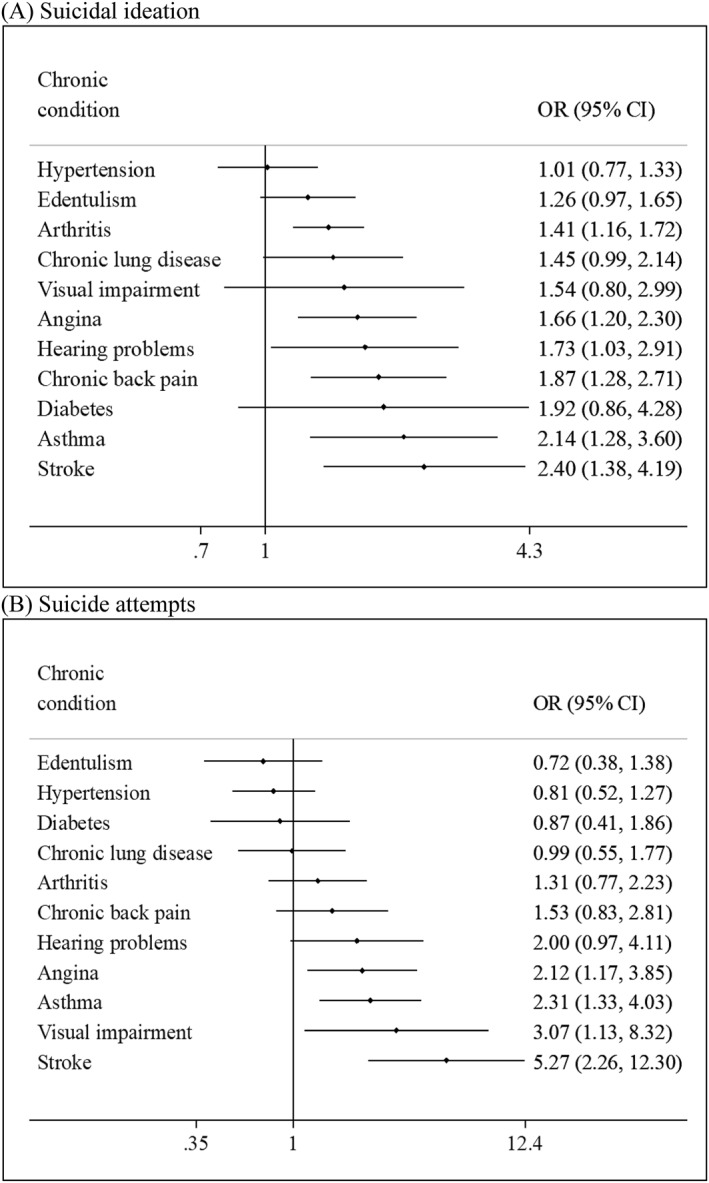
Association between individual chronic conditions and (A) suicidal ideation or (B) suicide attempts estimated by multivariable logistic regression. Models are mutually adjusted for all individual chronic conditions and age, sex, education, wealth, alcohol consumption, smoking, and country. Abbreviations: CI, Confidence interval; OR, Odds ratio

**FIGURE 3:**
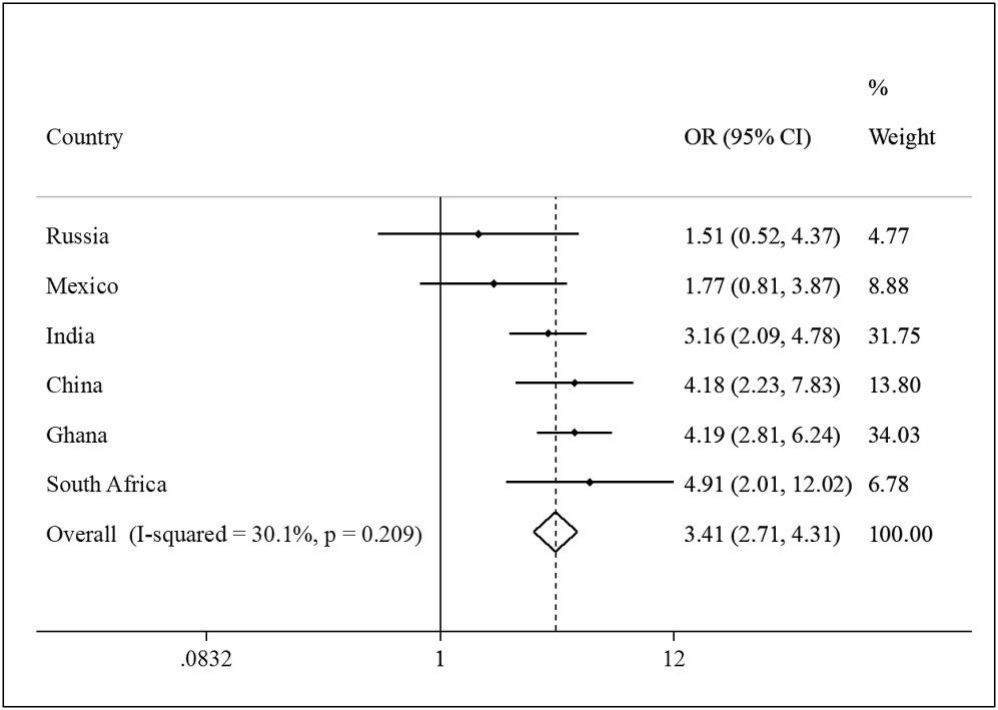
Country‐wise association between physical multimorbidity (i.e., ≥2 chronic conditions) and suicidal ideation (outcome) estimated by multivariable logistic regression. Models are adjusted for age, sex, education, wealth, alcohol consumption, and smoking. Overall estimate was obtained by meta‐analysis with fixed effects. Abbreviations: CI, Confidence interval; OR, Odds ratio

The final sample consisted of 34,129 adults aged ≥50 years. The mean (SD) age was 62.4 (16.0) years and 52.1% were females. The maximum age was 114 years. The sample size (% females) of each country was: China *n* = 13,175 (50.2%); Ghana *n* = 4305 (47.6%); India *n* = 6560 (49.0%); Mexico *n* = 2313 (53.2%); Russia *n* = 3938 (61.1%); South Africa *n* = 3838 (55.9%). The overall and sex‐wise prevalence of suicidal ideation, suicide attempts, individual chronic conditions, and multimorbidity (i.e., ≥2 chronic conditions) are shown in Table A3 of the Appendix. Overall, the prevalence of suicidal ideation, suicide attempts, and multimorbidity (i.e., ≥2 chronic conditions) were 3.4% (female 4.4%; male 2.2%), 0.6% (female 0.8%; male 0.4%), and 45.5% (female 50.2%; male 40.5%), respectively. Suicidal ideation and multimorbidity were both significantly associated with female sex, lower levels of wealth, unemployment, anxiety, loneliness, poor self‐rated health, as well as worse health status in terms of cognition, perceived stress, sleep/energy, mobility, and pain/discomfort (Table 1). The prevalence of suicidal ideation increased linearly with increasing numbers of chronic conditions, while for suicide attempts, the increase was less pronounced, and the prevalence was similar among those with 3 and ≥ 4 chronic conditions (Figure 1). After adjustment for potential confounders, arthritis, angina, hearing problems, chronic back pain, asthma, and stroke were associated with significantly higher odds for suicidal ideation, while the chronic conditions significantly associated with suicide attempts were angina, asthma, visual impairment, and stroke (Figure 2). In the overall sample, compared to no chronic conditions, 1, 2, 3, and ≥4 chronic conditions were associated with 1.77 (95% CI = 1.07–2.94), 2.72 (95% CI = 1.64–4.52), 3.90 (95% CI = 2.36–6.45), and 9.18 (95% CI = 4.46–18.88) times significantly higher odds for suicidal ideation, respectively (Table 2). For suicide attempts, 3 (OR = 6.15; 95% CI = 2.47–15.30) and ≥4 chronic conditions (OR = 5.39; 95% CI = 2.24–12.98) were associated with significantly higher odds. Interaction analysis showed that there is significant interaction by sex in the association between multimorbidity and suicidal ideation or suicide attempts with the OR being higher for males. Country‐wise analysis showed that multimorbidity is positively associated with suicidal ideation in all countries (i.e., OR>1) with the overall estimate based on a meta‐analysis being OR = 3.41 (95% CI = 2.71–4.31), with a low level of between‐country heterogeneity (*I* ^*2*^ = 30.1%) (Figure 3). Pain/discomfort (44.2%), sleep/energy (33.8%), and mobility (34.8%) were the main factors that explained the associated between multimorbidity and suicidal ideation (Table A4, Appendix), followed by poor self‐rated health (17.7%), cognition (11.3%), anxiety (10.1%), disability (6.9%), stress (6.4%), and loneliness (6.2%). Unemployment and social participation were not significant mediators. The mediated percentages were similar for suicide attempts although the figure was much higher for disability (13.9%) than in suicidal ideation.

**TABLE 1:** Sample characteristics (overall and by suicidal ideation or multimorbidity)

		Suicidal ideation				Multimorbidity^a^		
Characteristic		Overall	No	Yes	*P*‐value^b^	No	Yes	*P*‐value^b^
Age (years)	Mean (SD)	62.4 (16.0)	62.4 (16.0)	32.1 (16.0)	0.424	60.2 (14.4)	65.0 (16.7)	<0.001
Sex	Female	52.1	51.5	68.2	<0.001	47.6	57.3	<0.001
	Male	47.9	48.5	31.8		52.4	42.7	
Education (years)	Mean (SD)	6.0 (8.9)	6.1 (8.9)	4.3 (8.0)	<0.001	6.1 (9.1)	5.9 (8.9)	0.271
Wealth	Poorest	17.1	16.9	20.5	0.006	16.2	18.3	0.005
	Poorer	19.0	18.7	26.9		18.2	19.9	
	Middle	19.5	19.5	20.9		19.1	19.7	
	Richer	21.3	21.6	15.5		21.5	21.0	
	Richest	23.1	23.4	16.1		25.1	21.0	
Alcohol consumption	No	81.3	81.0	89.9	<0.001	80.3	82.6	0.081
	Yes	18.7	19.0	10.1		19.7	17.4	
Smoking	Never	58.6	58.7	53.0	0.122	57.0	59.9	<0.001
	Current	34.9	34.8	38.0		37.7	32.1	
	Past	6.6	6.5	9.0		5.4	8.0	
Unemployment	No	42.7	43.2	29.1	<0.001	50.5	33.0	<0.001
	Yes	57.3	56.8	70.9		49.5	67.0	
Anxiety	No	91.9	93.3	51.1	<0.001	94.7	88.3	<0.001
	Yes	8.1	6.7	48.9		5.3	11.7	
Loneliness	No	88.7	89.7	60.6	<0.001	91.9	84.6	<0.001
	Yes	11.3	10.3	39.4		8.1	15.4	
Disability	No	92.9	93.6	71.5	<0.001	97.3	87.5	<0.001
	Yes	7.1	6.4	28.5		2.7	12.5	
Self‐rated health	Not poor	78.2	79.2	50.3	<0.001	88.2	66.4	<0.001
	Poor	21.8	20.8	49.7		11.8	33.6	
Cognition^c^	Mean (SD)	30.6 (46.1)	29.9 (45.8)	49.1 (43.5)	<0.001	25.3 (44.1)	36.9 (46.7)	<0.001
Perceived stress^c^	Mean (SD)	40.1 (41.0)	39.6 (40.8)	55.1 (39.7)	<0.001	37.3 (40.8)	43.7 (41.1)	<0.001
Sleep/energy^c^	Mean (SD)	27.4 (45.2)	26.5 (44.7)	53.4 (37.9)	<0.001	19.9 (41.3)	36.6 (45.4)	<0.001
Mobility^c^	Mean (SD)	32.6 (46.6)	31.7 (46.3)	58.7 (36.6)	<0.001	23.7 (41.8)	43.4 (45.8)	<0.001
Pain/discomfort^c^	Mean (SD)	30.3 (44.9)	29.4 (44.4)	57.8 (35.5)	<0.001	22.4 (42.0)	39.9 (43.5)	<0.001
Social participation^d^	Mean (SD)	21.3 (23.3)	21.6 (23.2)	20.3 (22.8)	0.121	22.7 (23.7)	20.2 (22.8)	<0.001

*Note*: Data are % unless otherwise stated.

Abbreviation: SD, standard deviation.

^a^ ≥2 chronic conditions.

^b^ *p*‐value was calculated by Chi‐squared tests and Student's *t*‐tests for categorical and continuous variables, respectively.

^c^ Based on a scale ranging from 0 to 100 with higher scores representing worse health status.

^d^ Based on a scale ranging from 0 to 100 with higher scores representing higher levels of social participation.

**TABLE 2:** Association between number of chronic conditions or multimorbidity and suicidal ideation or suicide attempts (outcomes) estimated by multivariable logistic regression

		Suicidal ideation			Suicide attempts		
		Overall	Male	Female	Overall	Male	Female
No. of chronic conditions^a^	0	1.00	1.00	1.00	1.00	1.00	1.00
	1	1.77*	1.14	1.99*	1.64	0.48	2.34
		(1.07,2.94)	(0.52,2.49)	(1.04,3.84)	(0.64,4.25)	(0.11,2.10)	(0.67,8.13)
	2	2.72***	3.47**	2.33**	2.10	2.35	2.00
		(1.64,4.52)	(1.56,7.72)	(1.33,4.09)	(0.83,5.30)	(0.56,9.91)	(0.58,6.90)
	3	3.90***	6.52***	2.96***	6.15***	7.14***	5.78**
		(2.36,6.45)	(2.99,14.24)	(1.62,5.43)	(2.47,15.30)	(2.23,22.87)	(1.61,20.67)
	≥4	9.18***	13.40***	7.12***	5.39***	5.17**	5.65**
		(4.46,18.88)	(5.45,32.92)	(3.49,14.50)	(2.24,12.98)	(1.48,18.05)	(1.73,18.47)
Multimorbidity	No	1.00	1.00	1.00	1.00	1.00	1.00
(≥2 chronic conditions)	Yes	2.99***	5.82***	2.16***	2.79***	6.03***	2.08*
		(2.06,4.34)	(3.06,11.08)	(1.52,3.08)	(1.58,4.95)	(2.43,14.97)	(1.04,4.15)

*Note*: Data are odds ratio (95% confidence interval). Models are adjusted for age, education, wealth, alcohol consumption, smoking, and country. The analysis using the overall sample was additionally adjusted for sex.

^a^ Test for trend was significant for all six models (*p* < 0.05).

*p < 0.05, **p < 0.01, ***p < 0.001.

## 4 DISCUSSION

### 4.1 Main findings

In this large sample of middle‐aged and older age adults from six LMICs, it was observed that arthritis, angina, hearing problems, chronic back pain, asthma, and stroke were associated with significantly higher odds for suicidal ideation, while angina, asthma, visual impairment, and stroke were significantly associated with suicide attempts. In the overall sample, physical multimorbidity (i.e., ≥2 conditions) was associated with substantially increased odds for suicidal ideation (OR = 2.99; 95% CI = 2.06–4.34) and suicide attempts (OR = 2.79; 95% CI = 1.58–4.95). Interestingly, the association was observed to be stronger in males than females. Increasing numbers of chronic conditions dose‐dependently increased odds for suicidal ideation and suicide attempts with the OR (95% CI) for ≥4 chronic conditions (vs. no chronic conditions) being 9.18 (4.46–18.88) and 5.39 (2.24–12.98), respectively. Country‐wise analysis showed that there is a low level of between‐country heterogeneity in the association between multimorbidity and suicidal ideation. Cognition, anxiety, stress, sleep/energy, mobility, pain/discomfort loneliness, disability, and self‐rated health were identified as significant mediators.

### 4.2 Interpretation of the findings

Findings from the present study both support and add to previous literature. They support previous literature from high‐income countries and one LMIC (China) by confirming that a positive association exists between physical multimorbidity and suicidal ideation or suicide attempts.^7^, ^9^, ^32^, ^33^, ^34^ The present study adds to this literature by showing that such associations hold in a large sample of middle‐aged and older age adults from six LMICs.

The observed association between multimorbidity and suicidal ideation and suicide attempts may be explained by the cumulative impact of multiple individual chronic conditions. For example, angina, asthma, and stroke were all associated with increased odds for both suicidal ideation and suicide attempts. Angina may increase risk for suicidal behavior via autonomic nervous system dysfunction, inflammation pathways, cardiac arrhythmias, and altered platelet function.^35^, ^36^ Furthermore, in the case of asthma, distressing symptoms per se may increase risk for suicidality, while there may be a common genetic link leading to susceptibility to asthma and mood disorders (including suicidal behaviors).^37^ Finally, stroke was most strongly associated with suicidal ideation and suicide attempts in our study, and this may be explained by the psychological reaction to the disability associated with stroke, with suicide being regarded as a solution to a perceived intolerable life situation, as well as the psychological impact of dealing with cognitive and physical impairments, hopelessness, and other negative feelings.^38^

Apart from the cumulative impact of individual chronic conditions on suicidality, other factors may also explain the association. First, multimorbidity is associated with increased medication use to treat multiple conditions,^39^ and some medications may increase risk of suicidal thoughts and behavior (e.g., angiotensin receptor blockers).^40^ Second, household expenditure in relation to treat multiple chronic conditions is high, particularly in LMICs. Medicines are usually the largest component of costs, and this significant financial burden may increase risk for suicidality.^41^ Finally, the treatment of multimorbidity is complex, often requiring attendance to multiple treatment centers, and this complexity can increase feelings of hopelessness and worry that may lead to suicidality.^42^

Furthermore, the present study identified several potential mediators in the association of physical multimorbidity with suicidal ideation and suicide attempts. Pain/discomfort (mediated% 33.3%–44.2%), sleep/energy (28.2%–33.8%), and mobility limitations (26.6%–34.8%) explained the largest proportion of the association, while cognition, anxiety, stress, loneliness, disability, and self‐rated health explained this association to a lesser extent. Pain is common among those with multimorbidity,^22^ and pain has been observed to increase risk of suicidality via several mechanisms, including depression, hopelessness, desire for escape through death, and erosion of the natural fear of dying.^43^ Next, people with multimorbidity often experience sleep problems due to factors such as pain, difficulty breathing, and nocturia etc.^44^ In turn, sleep problems may increase risk for suicidal thoughts and behavior via mood regulation factors. For example, sleep may not provide an emotional refuge for distressed individuals, and thus, poor sleep quality may disrupt within‐sleep mood regulation processes.^45^

Multimorbidity is also often associated with mobility limitations due to factors such as pain and disability. This may increase risk for suicidality due to limitations in daily life, and mental health problems such as depression, anxiety, loneliness, and perceived stress.^46^ Finally, impairments in cognitive function have been reported in people with multimorbidity, and this may be explained by chronic conditions acting synergistically to accelerate cognitive decline as well as polypharmacy via the effect of drug interactions on cognition.^30^ In turn, cognitive decline (e.g., impairments in cognitive control, executive function, problem‐solving) make it difficult to cope with life problems functionally, and thus, can increase the risk of suicidal thoughts and behavior.^47^

An interesting finding in the present study is that the multimorbidity/suicidal ideation and suicide attempt relationship was observed to be stronger in males than females. Although the reason for this can only be speculated, this may be related with factors such as differences in disease profiles or likelihood of receiving antidepressants between women and men. Alternatively, in many LMICs, men are more likely to be the main wage earner, and having multiple chronic physical conditions may have a particularly detrimental effect on mental health in men for not being able to support the family financially. Moreover, suicidal thoughts and behavior tend to occur in conjunction with other health behaviors rather than alone.^48^ For example, suicidal behaviors are higher among illicit drugs users. Indeed, males are more likely to be illicit drug users than females.^49^ However, clearly more research is needed to understand the underlying reason for the gender difference observed.

### 4.3 Implications of the findings

Findings from the present study support the notion of screening for suicidality among people with physical multimorbidity in LMICs. This is particularly best placed to happen in the primary care setting as those with multimorbidity are particularly likely to be high utilizers of such services. During primary care visits, it may be prudent to implement either suicide‐specific self‐report measures or clinician interviews, including, for example, the Beck Scale for Suicide Ideation, Suicide Behaviors Questionnaire—Revised, and the Columbia Suicide Severity Rating Scale, among those with multimorbidity.^50^ If suicidal thoughts or behavior are subsequently identified, then it would be prudent to determine the underlying causes (e.g., pain, sleep problems) and address them or refer such individuals to mental health services or existing suicide prevention interventions. However, whether such screening and referral would be efficacious in LMICs where health systems are often fragmented is currently not known.

### 4.4 Strengths and limitations

The large sample of middle‐aged and older adults from multiple LMICs are clear strengths of the present paper. However, findings must be interpreted in light of their limitations. First, most of the information used in this study was based on self‐report, and therefore, social desirability and recall bias may exist. Moreover, it is possible that some questions such as those on suicidal ideation and suicide attempts were interpreted differently across different cultures. Second, the present measure of suicidal ideation was related to wish to die, which has been differentiated from active suicidal ideation. However, the presence of wishes to die has been reported as clinically important as the presence of active suicidal ideation. Third, suicidal ideation and suicide attempts were only assessed among those who had depressive symptoms (i.e., sadness, loss of interest or low energy). This may have led to an underestimation of those with suicidal ideation and suicide attempts, but it is worth noting that depressive symptoms are extremely common in people with suicidal behavior. Relatedly, due to this, it was not possible to assess the mediating effect of depression. Finally, because of the cross‐sectional nature of the study, causality or temporal associations cannot be established.

### 4.5 Conclusion

In this large sample of adults aged ≥50 years from six LMICs, physical multimorbidity was associated with an increased odds of suicidal ideation and suicide attempts, with a stronger association being observed in males than females. Future intervention and longitudinal cohort studies are warranted to assess whether addressing the mediators identified in our study can lead to reduction in suicidal ideation and suicide attempts in people with physical multimorbidity.

## CONFLICTS OF INTEREST

The authors declare that there is no conflict of interest.

## Data Availability

The data that support the findings of this study are available from the corresponding author upon reasonable request.

## Appendix

**TABLE A1:** Questions used to assess health status

Mobility	(1) Overall in the last 30 days, how much difficulty did you have with moving around?
	(2) In the last 30 days, how much difficulty did you have in vigorous activities, such as running 3 km (or equivalent) or cycling?
Pain and discomfort	(1) Overall in the last 30 days, how much of bodily aches or pains did you have?
	(2) In the last 30 days, how much bodily discomfort did you have?
Cognition	(1) Overall in the last 30 days, how much difficulty did you have with concentrating or remembering things?
	(2) In the last 30 days, how much difficulty did you have in learning a new task (for example, learning how to get to a new place, learning a new game, learning a new recipe etc.)?
Sleep and energy	(1) Overall in the last 30 days, how much of a problem did you have with sleeping, such as falling asleep, waking up frequently during the night or waking up too early in the morning?
	(2) In the last 30 days, how much of a problem did you have due to not feeling rested and refreshed during the day (e.g. feeling tired, not having energy)?
Perceived stress	(1) How often have you felt that you were unable to control the important things in your life?
	(2) How often have you found that you could not cope with all the things that you had to do?

**TABLE A2:** Details on the diagnosis of chronic conditions

Condition	(a) Self‐reported diagnosis	(b) Symptom‐based algorithm or other method of diagnosis^a^
Angina	Have you ever been diagnosed with angina or angina pectoris (a heart disease)?	Rose questionnaire^18^
Arthritis	Have you ever been diagnosed with/told you have arthritis (a disease of the joints, or by other names rheumatism or osteoarthritis)?	Affirmative answers to all four of the following: 1. During the last 12 months, have you experienced pain, aching, stiffness or swelling in or around the joints (e.g., in arms, hands, legs or feet) which were not related to an injury and lasted for more than a month? 2. During the last 12 months, have you experienced stiffness in the joint in the morning after getting up from bed, or after a long rest of the joint without movement? 3. Did this stiffness last for less than 30 min? 4. Did this stiffness go away after exercise or movement in the joint?
Asthma	Have you ever been diagnosed with asthma (an allergic respiratory disease)?	1. During the last 12 months, have you experienced attacks of wheezing or whistling breathing? (Yes) **AND** 2. “Yes” to at least one of the following (past 12 months): (a) Have you experienced an attack of wheezing that came on after you stopped exercising or some other physical activity? (b) Have you had a feeling of tightness in your chest? (c) Have you woken up with a feeling of tightness in your chest in the morning or any other time? (d) Have you had an attack of shortness of breath that came on without an obvious cause when you were not exercising or doing some physical activity?
Chronic lung disease	Have you ever been diagnosed with chronic lung disease (emphysema, bronchitis, COPD)?	1. During the last 12 months, have you experienced any shortness of breath at rest (while awake)? (Yes) **OR** 2. “Yes” to both of the following (past 12 months): (a) Have you experienced any coughing or wheezing for 10 min or more at a time? (b) Have you experienced any coughing up of sputum or phlegm on most days of the month for at least 3 months?
Diabetes	Have you ever been diagnosed with diabetes (high blood sugar)? (not including diabetes associated with a pregnancy)	NA
Hypertension	Have you ever been diagnosed with high blood pressure (hypertension)?	Blood pressure was measured three times with a one‐minute interval with the use of a wrist blood pressure monitor (Medistar Wrist Blood Pressure Model S) and the mean value of the three measurements was calculated. Hypertension was defined as having at least one of the following: Systolic blood pressure ≥140 mmHg; diastolic blood pressure ≥90 mmHg.
Stroke	Have you ever been told by a health professional that you have had a stroke?	NA

*Note*: For all chronic conditions, we assumed that the individual had the condition if they fulfilled at least one of the following: (a) affirmative answer to self‐reported diagnosis or (b) symptom‐based algorithm or other method of diagnosis.

^a^ These algorithms have been used in previous publications^16^, ^17^ and those of arthritis, asthma, and chronic lung disease have been validated.^16^, ^51^

**TABLE A3:** Prevalence of suicidal ideation, suicide attempts, and multimorbidity (overall and by sex)

Chronic condition	Overall	Female	Male	P‐value^a^
Suicidal ideation	3.4	4.4	2.2	<0.001
Suicide attempt	0.6	0.8	0.4	0.003
Angina	17.6	20.6	14.4	<0.001
Arthritis	29.5	34.5	24.2	<0.001
Asthma	7.9	7.0	8.8	0.003
Chronic back pain	8.6	11.1	5.9	<0.001
Chronic lung disease	15.8	15.2	16.4	0.139
Diabetes	6.8	7.1	6.6	0.390
Edentulism	12.9	14.3	11.5	0.002
Hearing problem	5.6	5.6	5.5	0.812
Hypertension	55.0	58.7	51.1	<0.001
Stroke	3.0	2.8	3.3	0.281
Visual impairment	1.3	1.6	0.9	0.001
Multimorbidity^b^	45.5	50.2	40.5	<0.001

*Note*: Data are %.

^a^ *P*‐value was calculated by Chi‐squared tests.

^b^ ≥ 2 chronic conditions.

**TABLE A4:** Mediators in the association between physical multimorbidity and suicidal ideation or suicide attempts (outcomes)

Outcome	Mediator	OR (95% CI)	*p*‐Value	OR (95% CI)	*p*‐Value	OR (95% CI)	*p*‐Value	% Mediated^a^
Suicidal ideation	Unemployment	2.99 (2.06,4.32)	<0.001	2.93 (2.04,4.20)	<0.001	1.02 (1.00,1.04)	0.072	NA
	Cognition	3.02 (2.08,4.39)	<0.001	2.67 (1.83,3.89)	<0.001	1.13 (1.08,1.18)	<0.001	11.3
	Anxiety	2.75 (1.86,4.08)	<0.001	2.48 (1.68,3.68)	<0.001	1.11 (1.08,1.14)	<0.001	10.1
	Stress	2.97 (2.07,4.26)	<0.001	2.77 (1.90,4.02)	<0.001	1.07 (1.04,1.11)	<0.001	6.4
	Sleep/energy	2.93 (2.04,4.22)	<0.001	2.04 (1.42,2.93)	<0.001	1.44 (1.33,1.55)	<0.001	33.8
	Mobility	2.97 (2.03,4.35)	<0.001	2.04 (1.35,3.08)	0.001	1.46 (1.34,1.59)	<0.001	34.8
	Loneliness	2.82 (1.94,4.11)	<0.001	2.65 (1.81,3.88)	<0.001	1.07 (1.04,1.09)	<0.001	6.2
	Social participation	3.00 (2.07,4.35)	<0.001	2.98 (2.06,4.32)	<0.001	1.01 (1.00,1.02)	0.127	NA
	Disability	2.84 (1.94,4.17)	<0.001	2.65 (1.80,3.90)	<0.001	1.07 (1.05,1.10)	<0.001	6.9
	Self‐rated health	2.92 (2.04,4.17)	<0.001	2.41 (1.74,3.35)	<0.001	1.21 (1.10,1.33)	<0.001	17.7
	Pain/discomfort	3.11 (2.11,4.60)	<0.001	1.89 (1.31,2.72)	0.001	1.65 (1.45,1.89)	<0.001	44.2
Suicide attempts	Unemployment	2.79 (1.57,4.96)	<0.001	2.73 (1.52,4.92)	0.001	1.02 (0.98,1.06)	0.291	NA
	Cognition	2.80 (1.57,4.99)	<0.001	2.54 (1.41,4.57)	0.002	1.10 (1.03,1.19)	0.009	9.5
	Anxiety	2.62 (1.45,4.74)	0.001	2.42 (1.32,4.44)	0.004	1.08 (1.04,1.13)	<0.001	8.3
	Stress	2.71 (1.51,4.85)	0.001	2.53 (1.40,4.57)	0.002	1.07 (1.01,1.13)	0.022	6.7
	Sleep/energy	2.76 (1.55,4.91)	0.001	2.07 (1.06,4.04)	0.032	1.33 (1.13,1.58)	0.001	28.2
	Mobility	2.75 (1.54,4.90)	0.001	2.10 (1.10,4.00)	0.024	1.31 (1.11,1.54)	0.001	26.6
	Loneliness	2.62 (1.44,4.77)	0.002	2.43 (1.33,4.44)	0.004	1.08 (1.04,1.12)	<0.001	8.0
	Social participation	2.79 (1.57,4.96)	<0.001	2.81 (1.58,4.99)	<0.001	1.00 (0.98,1.01)	0.505	NA
	Disability	2.54 (1.38,4.70)	0.003	2.23 (1.20,4.15)	0.011	1.14 (1.09,1.19)	<0.001	13.9
	Self‐rated health	2.68 (1.48,4.84)	0.001	2.21 (1.17,4.20)	0.015	1.21 (1.10,1.33)	<0.001	19.2
	Pain/discomfort	2.80 (1.57,4.97)	<0.001	1.98 (1.01,3.89)	0.046	1.41 (1.13,1.75)	0.002	33.3

*Note*: Models are adjusted for age, sex, education, wealth, alcohol consumption, smoking, and country.

Abbreviations: OR, odds ratio; CI, confidence interval.

^a^Mediated percentage was only calculated in the presence of a significant indirect effect (*p* < 0.05).
